# Commentary: Alpha_1_-adrenergic receptor blockade in the ventral tegmental area attenuates acquisition of cocaine-induced pavlovian associative learning

**DOI:** 10.3389/fnbeh.2023.1147507

**Published:** 2023-02-23

**Authors:** Anna Lasne, Merkourios Simos, Loris Constantin, Brian D. McCabe, Carmen Sandi

**Affiliations:** ^1^Life Sciences Engineering Master Program, School of Life Sciences, Ecole Polytechnique Fédérale de Lausanne (EPFL), Lausanne, Switzerland; ^2^Brain Mind Institute, School of Life Sciences, Ecole Polytechnique Fédérale de Lausanne (EPFL), Lausanne, Switzerland

**Keywords:** cocaine, ventral tegmental area (VTA), phasic dopamine, alpha 1-adrenergic receptor, reward learning, associative learning, salience

The ability to adapt to environmental rewards is crucial for survival. Animals must identify rewards and direct their behavior to acquire them, while differentiating aversive stimuli (Berridge, 2000; Roitman et al., 2008). Extensive research has attributed this process to the phasic release of dopamine (DA) in the forebrain (Wise, 2004; Schultz, 2007; Saunders et al., 2018). However, while rapid DA release from ventral tegmental area (VTA) neurons projecting into the nucleus accumbens (NAc) have been regarded as the principal mechanism of reward-based learning (Robbins and Everitt, 1996; Glimcher, 2011; but see Jeong et al., 2022), emerging evidence suggests that noradrenaline (NA) actions in the mesolimbic system are also involved in reward processing (Jahn et al., 2018); a possibility that was initially discounted (Davis et al., 2022).

Out of the three classes of adrenergic receptors, alpha-1 (α1-AR), alpha-2 (α2-AR), and beta-adrenergic receptors (β-AR), α1-ARs have been implicated in the regulation of dopamine release in the VTA-NAc pathway (Grenhoff and Svensson, 1993; Grenhoff et al., 1995; Paladini and Williams, 2004; Rommelfanger et al., 2009), affecting cue-reward associations (Mitrano et al., 2012). Early work showed that α1-ARs interact with cocaine (Drouin et al., 2002; Hyman et al., 2006), leading to addiction due to the blockade of DA and NA uptake by neuronal plasma membrane transporters (Kuhar et al., 1991). Targeting α1-ARs could, therefore, be a promising strategy to counteract the behavioral symptoms of cocaine addiction. However, the complex relationship between cocaine-induced effects and reward-learning processes has hampered the development of effective treatments (Thomas et al., 2008; Buchholz and Saxon, 2019) and revealed two important knowledge gaps. First, while adrenergic receptors are expressed in the VTA (Solecki et al., 2017; Kielbinski et al., 2019), the exact regulatory function of VTA α1-ARs was not well-understood. Second, although previous work studied α1-ARs in the context of cocaine-seeking behavior (Zhang and Kosten, 2005; Flagel et al., 2009; Rommelfanger et al., 2009; Solecki et al., 2018; Schutte et al., 2020), whether α 1-AR antagonists can affect cocaine-induced reward cue salience, a crucial component of drug addiction, had yet to be established.

In their recent work, Solecki et al. (2022) addressed these two questions by using prazosin, an α1-AR antagonist, to investigate whether α1-ARs affect cue salience in the cocaine-based conditioned place preference (CPP) paradigm. The CPP paradigm has been used repeatedly to study cocaine's effect on reward perception (Calcagnetti et al., 1995; Caffino et al., 2021). However, this is the first time that results are directly contrasted with the outcome of the same experimental manipulation in an instrumental learning task, enabling the distinction between salience and valence. Additionally, the study included the measurement of ultrasound vocalization (USV) and of NAc dopamine levels.

The research shows that prazosin administration into the VTA attenuates the acquisition of cocaine-induced CPP, suggesting that α1-AR blockade impairs associative learning. Significantly, the α1-AR antagonist did not affect locomotion or induce a stand-alone effect in CPP. These results confirmed that α1-ARs have a purely modulatory role in encoding cue salience under cocaine influence.

To assess whether CPP inhibition by prazosin was due to an induced insensitivity to reward, the authors performed an instrumental learning task, measuring the rate of cocaine self-administration, with and without α1-AR blockade in the VTA. They reported no difference between the two conditions, indicating that α1-ARs are not involved in reward sensitivity and, therefore, isolating cue salience as the main effect of NA. This conclusion was further validated by the finding that prazosin had no effect on cocaine-induced USVs, indicating no change in the cocaine-induced positive affective state.

Immunohistochemical analyses demonstrated that α1-ARs in the VTA are primarily expressed in TH-positive neurons. However, α1-ARs were also partially colocalized with GABAergic interneurons and astrocytes. Their expression in these additional cell types may also be implicated in NA signaling, a possibility that will need to be addressed by future studies. Additionally, the authors analyzed phasic dopamine release following intra-VTA prazosin administration, revealing the attenuation of dopamine release by the antagonist in parallel to inhibiting Pavlovian learning. These results solidify the functional role of VTA α1-ARs in associative learning, *via* the modulation of downstream DA signaling.

Nevertheless, future work is required to fully understand the complexity of cocaine effects. For example, the authors found a striking non-linear dosage effect of cocaine-induced CPP. Increasing cocaine dosage from 20 to 25 mg/kg completely extinguished the acquisition of CPP, producing a CPP score equivalent to the control test of saline administration. Additionally, recent work has demonstrated that cocaine can directly affect NA levels through protein kinase C signaling (Zhu et al., 2022). Together, these results indicate that the effect of α1-AR blockade may be more complex than simply inhibiting the encoding of cue saliency. For instance, an increase or decrease in NA uptake due to α1-AR activity modulation could hypothetically produce the same outcome of diminished CPP. Consequently, a crucial next step would be to perform a rigorous analysis of the interactions between α-adrenergic drugs and cocaine, as a pre-requisite to further considering α-adrenergic drugs as potential therapeutic drugs in cocaine addiction.

To understand potential causes of non-linear cocaine dosage effects, a systematic study of cocaine release could be informative. Recently-developed optical sensors could be used to compare NAc DA levels following cocaine administration in absence and presence of VTA α1-AR blockade (Patriarchi et al., 2018). Additionally, α1-AR agonists – formerly utilized in cocaine behavioral studies (Schmidt et al., 2017) – could be used to provide further insights into the role of adrenergic receptors in reward learning. Furthermore, previous work has associated learning impairments with intraperitoneal administration of prazosin independently of cocaine administration (Stuchlík et al., 2009). These findings warrant more stringent controls to ensure that observed behavioral changes are caused by the interaction between cocaine and prazosin, and not due to stand-alone effects of prazosin administration.

In summary, the work by Solecki et al. (2022) is a welcome addition to a growing body of studies investigating the various roles of NA in shaping behavior, in the context of drug addiction (Smith and Aston-Jones, 2011; Perry et al., 2015). The next challenge is to integrate these novel findings into a comprehensive understanding of the role of NA in reward-based learning. While our understanding of the mesolimbic pathway is far from complete, the development of quantitative models incorporating knowledge from both DA and NA systems should provide a more accurate view of reward learning, guiding new insights into the mechanisms of reward and the treatment of addiction.

## Author contributions

AL and MS wrote the first draft. AL, MS, LC, BM, and CS edited and contributed to the writing of the final draft. All authors contributed to the article and approved the submitted version.
